# The dentary of *Australovenator wintonensis* (Theropoda, Megaraptoridae); implications for megaraptorid dentition

**DOI:** 10.7717/peerj.1512

**Published:** 2015-12-15

**Authors:** Matt A. White, Phil R. Bell, Alex G. Cook, Stephen F. Poropat, David A. Elliott

**Affiliations:** 1 Mechanical Engineering, University of Newcastle, Callaghan, New South Wales, Australia; 2 Palaeontology, Australian Age of Dinosaurs Museum of Natural History, Winton, Queensland, Australia; 3 School of Environmental and Rural Science, University of New England, Armidale, New South Wales,Australia; 4 School of Earth Science, University of Queensland, St Lucia, Queensland, Australia

**Keywords:** Australovenator wintonensis, Winton formation, Theropod, Cretaceous, Dentary, Teeth, Australian dinosaurs, Megaraptorid

## Abstract

Megaraptorid theropods were an enigmatic group of medium-sized predatory dinosaurs, infamous for the hypertrophied claw on the first manual digit. Megaraptorid dentition is largely restricted to isolated teeth found in association with skeletal parts; however, the *in situ* maxillary dentition of *Megaraptor* was recently described. A newly discovered right dentary pertaining to the *Australovenator* holotype preserves *in situ* dentition, permitting unambiguous characterisation of the dentary tooth morphology. The new jaw is virtually complete, with an overall elongate, shallow profile, and fifteen visible *in situ* teeth at varying stages of eruption. *In situ* teeth confirm *Australovenator* exhibited modest pseudoheterodonty, recurved lateral teeth with a serrate distal carina and reduced mesial carina, similar to other megaraptorids. *Australovenator* also combines of figure-of-eight basal cross-section with a lanceolate shape due to the presence of labial and lingual depressions and the lingual twist of the distal carina. Computed tomography and three-dimensional imagery provided superior characterisation of the dentary morphology and enabled an accurate reconstruction to a pre-fossilised state. The newly established dental morphology also afforded re-evaluation of isolated theropod teeth discovered at the *Australovenator* holotype locality and from several additional Winton Formation localities. The isolated Winton teeth are qualitatively and quantitatively similar to the *in situ* dentary teeth of *Australovenator*, but are also morphometrically similar to Abelisauridae, Allosauridae, Coelophysoidea, Megalosauridae and basal Tyrannosauroidea. Qualitative characters, however, clearly distinguish the teeth of *Australovenator* and the isolated Winton teeth from all other theropods. Evidence from teeth suggests megaraptorids were the dominant predators in the Winton Formation, which contrasts with other penecontemporaneous Gondwanan ecosystems.

## Introduction

**Figure 1:**
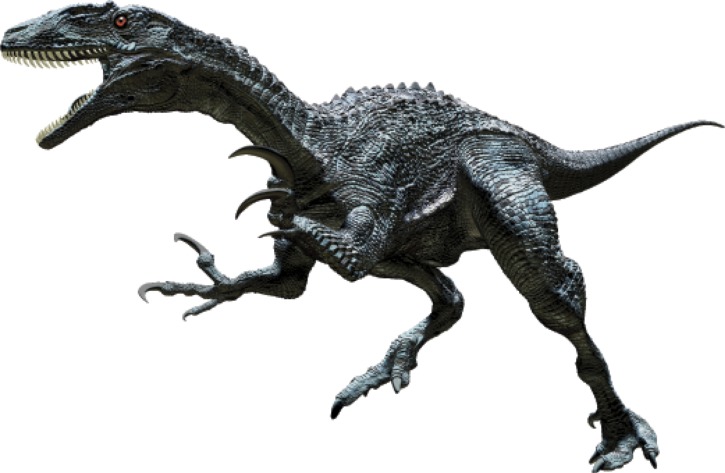
Reconstruction of *Australovenator wintonensis.* Artwork created by Travis R. Tischler.

*Australovenator wintonensis*Hocknull et al., 2009 holds the distinction as Australia’s most complete theropod dinosaur comprising of mostly forearm (White et al., 2012) and hind limb elements (White et al., 2013a) (Fig. 1). The majority of these specimens were discovered and described following the holotype description as preparation of concretions from the holotype locality is ongoing. Newer elements continue to broaden our understanding of megaraptorid morphology. Herein we describe a newly discovered right dentary of the *Australovenator* holotype specimen AODF (Australian Age of Dinosaur Fossil) 604. The right dentary is better preserved than the left and provides new information on megaraptorid lower jaw morphology, which is otherwise poorly known across Megaraptoridae (Novas, Ezcurra & Lecuona, 2008; Porfiri et al., 2014). Megaraptoridae (sensu Novas et al., 2013) comprises of predominantly Gondwanan theropods: *Aerosteon riocoloradensis* Sereno et al., 2008, *Megaraptor namunhuaiquii* Novas, 1998 and *Orkoraptor burkei* Novas, Ezcurra & Lecuona, 2008 from South America as well as *Australovenator wintonensis* and a second unnamed taxon from Australia (Bell et al., 2015). *Eotyrannus lengi* Hutt et al., 2001 from Europe hints at a putatively wider but equivocal occurrence for Megaraptoridae.

A phylogenetic re-evaluation of *Australovenator* is still premature as preparation of holotype material is ongoing. Nevertheless, the new *Australovenator* dentary retains *in situ* dentition, which has implications for the identification of isolated megaraptorid teeth particularly within the Winton Formation.

Ten isolated theropod teeth were discovered alongside the *Australovenator* holotype along with a partial sauropod skeleton (Hocknull et al., 2009; Poropat et al., 2014). Three additional localities in the Winton Formation have also produced an isolated shed theropod tooth in association with sauropod remains. All of these teeth were re-evaluated using a combined morphological and multivariate statistical approach in order to better understand their affinities and potential theropod diversity in the Late Cretaceous of central Queensland.

## Methods

### Specimen preparation

The right dentary was prepared using pneumatic air scribes and consolidated with Paraloid B72. Polyethylene Glycol PEG 3350 ‘Carbowax’ was used to support fragile specimens during preparation, filling gaps and cracks, providing support and helping absorb vibration from pneumatic preparation tools.

### Computed tomography

Computed tomographic (CT) scans of both dentaries were conducted at Queensland Xray, Mackay Mater Hospital, central eastern Queensland using a Brilliance CT 64-channel scanner (Koninklijke Philips N.V) capable of producing 0.9 mm slice images. Mimics version 10.01 software (Materialise HQ, Leuven, Belgium) was used to view and reconstruct internal structures of the dentary, enabling images to be scrolled though in sequence in each aspect view to better visualise internal structures.

### Three-dimensional reconstruction

Mimics 10.01, was used to delineate various structures within both dentaries. Separate meshes were developed of the broken sections, erupted teeth, newly forming enamel and resorption pits. 3D PDFs (see Figs. S1–S3) were assembled from objects generated in Mimics (vers. 16.02) using Adobe Acrobat XI Pro and Adobe 3D PDF Converter 4.1.

**Figure 2:**
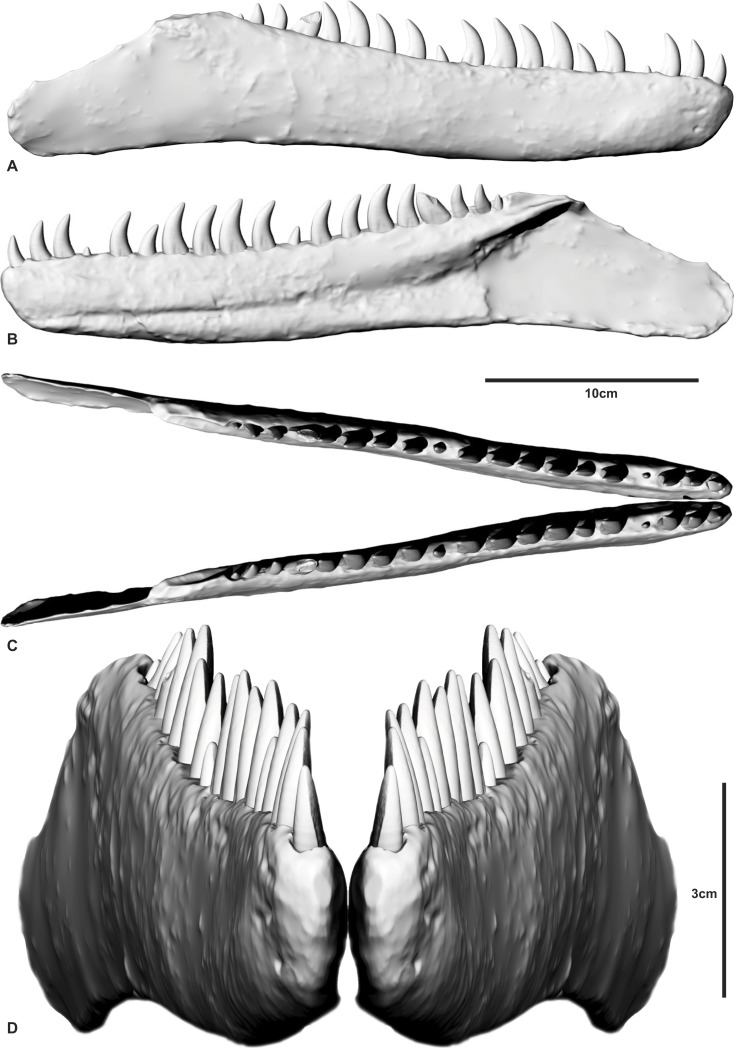
Reconstructed dentary of *Australovenator wintonensis* by Travis R. Tischler. (A) Labial;(B) Lingual; (C) Cranial; (D) Anterior.

The meshes were exported as Binary STL files into Rhinoceros 4.0 (Robert McNeal & Associates, Seattle, WA, USA). The broken sections of the dentary were realigned to their correct position using the internal structures as guides. The rotate and move tools under the ‘Transform’ menu was used to accomplish the realignment. To digitally repair the right dentary, the realigned mesh was imported into Zbrush 4R6 Pixologic as an OBJ file. The file was appended as a sub-tool Zsphere to create a 3D polymesh in the same space as the imported scan file. Located within the sub-tool menu a projection tool (outer) is used to project the detail from the scan file onto the new mesh. The ‘outer’ projection of the mesh projected the mesh to the outer surface but no further which filled in the post-mortem fractures on the specimen. Most of the right *in situ* dentary teeth were poorly preserved and had their apical tips missing. To restore these teeth we used the mesh developed from a micro CT scan of a near perfectly preserved isolated theropod tooth AODF826 discovered at the Matilda site to re-tooth the dentary. The *in situ* dentary teeth were used as a guide to achieve the correct tooth proportions and eruption stage within each socket (Fig. 2).

### Images

Tooth photographs and measurements were taken with a Dino-Lite Premier microscope. The associated software DinoCapture 2.0 (Version 1.4.1) enabled measurements to be taken from the captured photograph. The Dino-Lite was calibrated with a 5 mm scale bar prior to use.

## Geology

**Figure 3:**
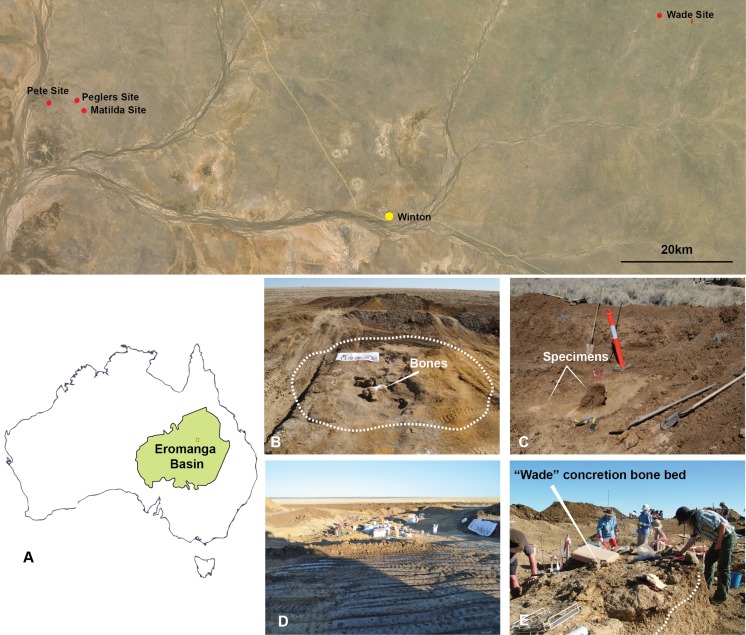
Australian Age of Dinosaur Localities of *Australovenator* holotype and isolated theropod teeth. (A) Topographic map of all five localites and their relative position within the Eromanga Sedimentary Basin: Matilda Site (AODL 85), Pete Site (AODL 125), Pegler’s Site (AODL 124), and Wade Site (AODL 82); (B) Holotype quarry of *Australovenator* Matilda Site; (C) Pegler’s Site; (D) Pete Site; (E) Wade Site.

*Australovenator* was discovered in Cenomanian (ca. 95 Ma) deposits of the Winton Formation on Elderslie Station near the town of Winton, central-western Queensland (informally referred to as the ‘Matilda site’; Australian Age of Dinosaurs Locality (AODL85) (Fig. 3). The site was excavated over five field seasons, which yielded hundreds of disarticulated bones pertaining to *Australovenator* and the titanosaur *Diamantinasaurus*; however, the association between the two dinosaurs has not yet been addressed. Three additional isolated theropod teeth were discovered at three separate localities alongside presently undescribed sauropod bones all within several tens of kilometres of AODL85. The remains from these sites were recovered from modern black soil with no distinctive facies, although all were undoubtedly derived from the Winton Formation, which underlies the modern pedogenic horizon. The geology at AODL85 has been interpreted as representing an oxbow lake deposited near the eastern margin of the cool, epicontinental Eromanga Sea (Hocknull et al., 2009; Rey, 2013; White et al., 2013b). These beds were laid down at a palaeolatitude of approximately 51°S (Seton et al., 2012). The bone-bearing layer consists of a bluish-grey claystone rich in plant material. Superimposing sand beds on the bone-bearing clay was sampled and revealed a detrital zircon age of Cenomanian age (ca 95 Ma) (Bryan et al., 2012). Detrital zircon ages for the Winton Formation record a depositional history from ∼103 to 92 Ma (Tucker et al., 2013).

## Multivariate Statistics and Terminology

The *in situ* teeth of the holotype (AODF604) and isolated teeth from all four Winton Formation localities were assessed for the following: crown base length (CBL, Hendrickx, Mateus & Araújo, 2015; FABL of some authors), measured at the base of the crown from its mesial-most to its distal-most extension; crown base width (CBW, Hendrickx, Mateus & Araújo, 2015), the labio-lingual extension of the crown at its base; crown height (CH, Smith, 2005), the apicobasal height measured perpendicular to the CBL to the highest point on the tooth; crown tooth angle (CTA), the angle between lines drawn along the level of the CBL and the apical tip of the tooth, and; crown height ratio (CHR) defined as the CH divided by CBL (Table 1).

**Table 1:** Specifications of isolated theropod teeth from the Winton Formation. Abbreviations: NP, Not preserved; CH, Crown height; CBL, Crown base length; CBW, Crown base width; WOA, Wear on apex; MC, Denticles on mesial surface per millimetre; DC, Denticles on distal surface per millimetre; CTA, Crown tooth angle; ILN, Informal locality name; LN, Locality number, [2] second tooth in the right dentary (AODF 604).

Specimen number	CH	CBL	CBW	WOA	DMS	DDS	CTA	CH ratio	ILN	LN
AODF 831	12.5	7.3	4.8	YES	–	3	63	1.71	Matilda site	AODL 85
AODF 822	14	6.8	6	YES	3	3	62	2.0	Matilda site	AODL 85
AODF 823	13.6	9.3	5.5	NO	–	3	46	1.42	Matilda site	AODL 85
AODF 824	12.8	8.4	4.3	NO	–	3	51	1.52	Matilda site	AODL 85
AODF 825	18.4	10	6.2	NO	2	3	61	1.62	Matilda site	AODL 85
AODF 826	16.5	9.5	6.5	NO	–	3	61	1.73	Matilda site	AODL 85
AODF 829	16.4	10.4	5.7	NO	–	3	50	1.57	Matilda site	AODL 85
AODF 828	14.4	9.4	5.8	YES	–	3	53	1.53	Matilda site	AODL 85
AODF 827	19.8	9.7	NP	YES	NP		58	2.04	Matilda site	AODL 85
AODF 830	16.3	11.7	NP	–	NP	NP	55		Matilda site	AODL 85
AODF 664	9	5.3	4.1	YES	–	3	61		Pegler’s site	AODL 124
AODF 820	16.5	9.7	7.2	NO	3	3	53	1.71	Pete site	AODL 125
AODF 819	17.8	8.3	10.2	NP	–	2	58		Wade site	AODL 82
AODF 604 [2]	*17.5	*9.1	*5.3	–	?	4	NP	NP	Matilda site	AODL 85

Mesial denticle density (MC) and distal denticle density (DC)—both measures of the number of denticles per millimetre at mid crown height—were recorded. Descriptions of dental morphology follow the terminology recently defined by Hendrickx, Mateus & Araújo (2015). *In situ* teeth are referred to by their position in the dentary (D), thus the fourth dentary tooth is D4, whereas the eleventh tooth is D11.

In order to quantitatively evaluate the relationships between the teeth of *Australovenator* and the isolated theropod teeth from the Winton Formation with other theropods, our dataset was added to a modified version of the recent comprehensive dataset of Hendrickx, Mateus & Araújo (2014), which includes 995 theropod teeth assigned to 18 taxonomic groups corresponding to Coelophysoidea, Noasauridae, Abelisauridae, Megalosauridae, Spinosauridae, Allosauridae, Neovenatoridae, Carcharodontisauridae, Tyrannosauridae, Dromaeosauridae, Troodontidae, as well as paraphyletic groupings for non-neotheropod Theropoda, non-abelisauroid Ceratosauria, and non-tyrannosaurid Tyrannosauroidea (see Hendrickx, Mateus & Araújo, 2014 for the source of data collected from other authors).

As per the original analysis, several theropods with uncertain affinities (*Erectopus, Nuthetes, Piatnitzkysaurus, Richardoestesia*) were analysed at the genus level. Modifications to the dataset of Hendrickx, Mateus & Araújo (2014) include, (1) the removal of specimens formerly identified as *Australovenator* (which are here considered part of the isolated Winton Formation tooth dataset (see below)) from Neovenatoridae (sensu Benson, Carrano & Brusatte, 2010), which includes only the teeth of *Neovenator*; (2) the placement of *Aerosteon* and *Fukuiraptor* into a separate clade, Megaraptora, and; (3) the addition of 27 teeth corresponding to the holotype dentary of *Australovenator* and isolated teeth from the Winton Formation (herein, simply referred to as the isolated Winton teeth), which were separated into two additional categories. Because missing data can significantly influence the results of morphometric analyses, variables with a large proportion of missing data (>35%) for all taxa (apical length, mid-crown length, mid-crown width, and mid-crown ratio (see Hendrickx, Mateus & Araújo, 2015 for definitions) in the original dataset were also omitted. Furthermore, *in situ* teeth D5, D11 and a single shed tooth, AODF819, were omitted from the statistical analysis because of extensive diagenetic damage (AODF819 and *in situ* tooth D11), or because it was incompletely erupted (*in situ* tooth D5). All of these were interpreted as imprecisely reflecting the actual crown morphology (Table 2). As per Hendrickx, Mateus & Araújo (2015), a discriminant analysis (=canonical variate analysis) was performed in order to assess whether *Australovenator* and the isolated Winton teeth could be identified and differentiated from other theropods based on quantitative data. In total, 1,022 teeth corresponding to 21 different clades were analysed and seven measured variables (CBL, CBW, CH, MC, DC, CBR, and CHR) were analysed in the statistical software PAST3 Hammer, Harper & Ryan (2001). All data was log transformed to more closely resemble a normal distribution for all measurements (see Samman et al., 2005). Where denticles were absent (e.g., Spinosauridae, *Australovenator*), MC and DC were coded as ‘?’. Unknown values were coded as ‘?’. In order to better visualise teeth that were found to be morphometrically similar to *Australovenator* and the Winton teeth, a second discriminant analysis was performed on a reduced dataset (herein, the reduced taxa dataset) that excluded clearly differentiable morphotypes (as revealed by the first analysis) corresponding to Spinosauridae, Troodontidae, *Nuthetes*, and *Richardoestesia*.

**Table 2:** Select measurements for in situ dentary teeth of the holotype Australovenator wintonensis used in the multivariate analysis. Measurements in millimetres. Abbreviations: PCH, Preserved crown height; CBL, Crown base length; CBW, Crown base width; CHR, Estimated reconstructed crown height; CTA, Estimated crown tooth angle.

AODF 604ALVEOLI NUMBER	PCH	CBL	CBW	Base tooth angle	CHR	CTA
1	9.5	7.4	59	–	13.5	57
2	9.6	9.1	5.3	–	17.5	68
4	0.8	–	–	63	3.8	–
5	5.8	9.8	6.8	–	20.3	63
6	7.9	8.3	4.4	51	13.9	57
7	9.6	10.1	5.5	–	22.8	65
8	12.7	9.9	4.6	65	21	59
9	13.8	10.5	6.2	–	23.4	64
10	12.21	9.8	6.3	68	21.5	59
11	5.5	4.7	4.1	71	7.6	–
13	14.4	8.9	4.8	64	18.2	51
14	11.1	10.8	5.5	–	21.5	56
15	13.5	9.7	4.6	55	18.1	58
16	16.5	11.4	4.9	60	13.6	–
17	11.2	8.7	4.1	–	11.9	62
18	10.9	7	4.3	72	10.4	47
19	–	–	–	–	2.7	–

Because most of the *in situ* teeth in the holotype dentary of *Australovenator* were broken, a second set of analyses was performed, which supplemented actual CH for estimated crown height (RCH; reconstructed using the methods described above). Crown height ratio (CHR) was recalculated from the new values whereas all other variables remained unchanged. Two discriminant analyses were performed on this dataset; the first, taking into account all 21 morphotypes, was followed by a second discriminant analysis on a reduced taxa dataset as per the first set of analyses.

### Ethics statement

All necessary permits were obtained for the described study, which complied with all relevant regulations. Permission to excavate the specimens from Elderslie Station was obtained from the landholders. During excavation each specimen was given a temporary field number for location and storage purposes. Specimens were donated by the landholders to the Australian Age of Dinosaurs Museum of Natural History (AAOD) where they were finally prepared and formally identified. All specimens pertaining to the holotype *Australovenator wintonensis* are allocated the specimen number AODF604 and stored in a climate controlled type room at the AAOD, 15 km east of Winton, Queensland, Australia.

**Figure 4:**
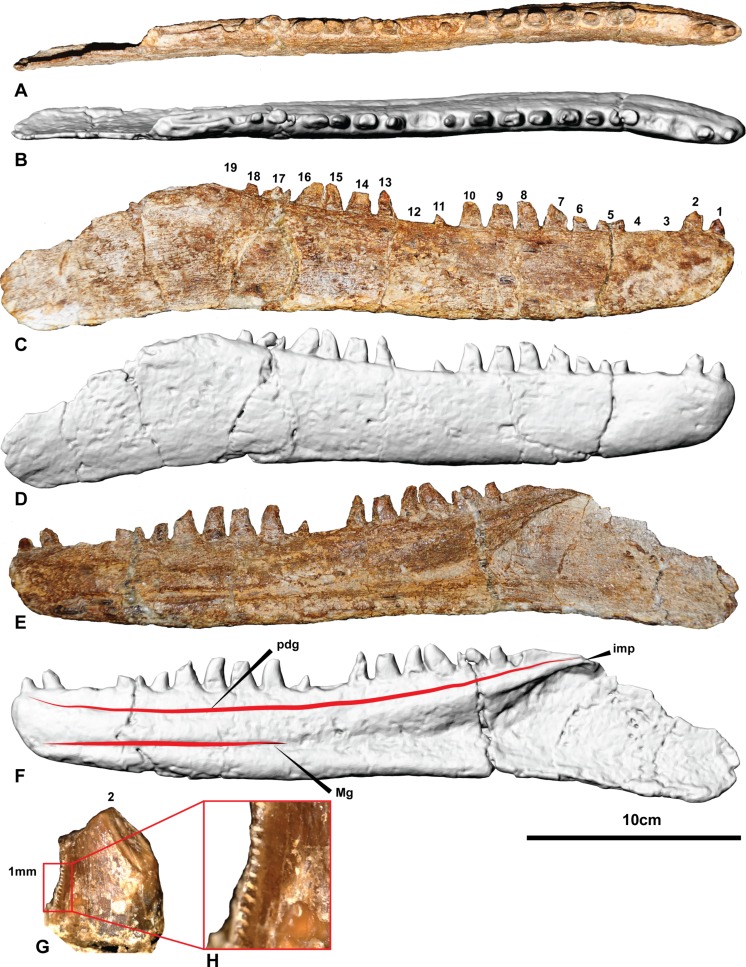
The holotype right dentary of *Australovenator wintonensis* AODF604. Photographs in: (A) Dorsal; (C) Labial; (E) Lingual. Digital renders in: (B) Dorsal; (D) Labial; (F) Lingual; (G) second tooth of the right dentary preserving denticles; (H) close up of denticles. Abbreviations: dc, distal carina; imp, intramandibular process of dentary; ld, lateral depression; lab, labial depression; pdg, paradental groove; Mg, Meckelian groove. Scale bar = 10 cm.

## Results

### Right dentary (AODF604)

The right dentary is complete with 15 visible entire and partial teeth preserved within the 19 alveoli (Fig. 4 and Fig. S1). Unlike the left dentary, the right is relatively uncrushed. In general, the dentary is slender and elongate measuring 342.6 mm long. In lateral view, the anterior end is rounded and the ventral margin is weakly sinuous. The alveolar margin and the ventral margin of the dentary are parallel anteriorly, becoming divergent posterior to about the twelfth alveolus. Posteriorly, the dentary is extremely thin (1–2 mm) where it contacted the surangular. The intermandibular symphysis is not distinguishable on the right dentary (*contra* Hocknull et al., 2009). The symphysis is small and poorly-defined in *Fukuiraptor*, *Eotyrannus* (see Fig. 3 in Hutt et al., 2001) and *Neovenator* (see Text-Fig. 8 in Brusatte, Benson & Hutt 2008).

In dorsal aspect the dentary is relatively straight, although fractures through alveoli 5 and 17 have caused an unnatural anterolateral bend in the symphyseal region. These fractures also transversely sheared the respective tooth crowns out of correct alignment.

Medially, a shallow paradental groove occurs immediately ventral to the alveolar margin, which dorsally outlines a thickened medial band. More ventrally, the Meckelian groove originates proximal to the nineteenth alveolus where it is tallest and deepest. It tapers anteriorly, becoming a dorsoventrally narrow groove between alveolus 11 and the symphysis where it is situated low in the dentary. The groove terminates at the anterior tip of the dentary just ventral to the presumed symphyseal facet. A Meckelian foramen ventral to alveolus 5, as reported by Hocknull et al. (2009), could not be confirmed in either left or right dentaries.

A distinct medial band and the Meckelian groove are also present in *Neovenator* (Brusatte, Benson & Hutt, 2008); however, the medial dentary surface of *Eotyrannus* was described as relatively flat (Hutt et al., 2001).

A row of primary neurovascular foramina and secondary neurovascular foramina on the lateral surface was initially reported on the left dentary (Hocknull et al., 2009); however, the better-preserved right dentary is smooth along its lateral surface with no distinct primary or secondary neurovascular foramina. Their presumed presence in the left dentary (Figs. S2 and S3) appears to be the result of poor preservation as the surface veneer was not preserved. Their apparent absence is unusual and demonstrates a potential autapomorphic feature of *Australovenator*.

The interdental plates are difficult to distinguish in both left and right dentaries due to poor preservation and ironstone covering. There is no distinction between the plates and the jaw bone indicating the plates were fused. Interestingly, the dentary of *Eotyrannus* is described as possessing interdental plates resembling small spikes that project between the alveoli (Hutt et al., 2001). However, it was elaborated that these spikes could not be differentiated from bone on the dentary’s labial surface and resembled the lingual alveolar margin of *Deinonychus antirropus* Ostrom 1969 (Hutt et al., 2001). The basal megaraptoran *Fukuiraptor* was described as possessing fused interdental plates (see Fig. 3 in Azuma & Currie, 2000).

Emergent teeth are visible in all alveoli except D3, D4, D12 and D19, and all but D11, D15, and D16 have their apical tips missing. In general, all except the first tooth are ziphodont, recurved, bladelike, with a rounded mesial edge and pointed distal edge (corresponding to the distal carina) in cross-section (at mid crown height).

**Figure 5:**
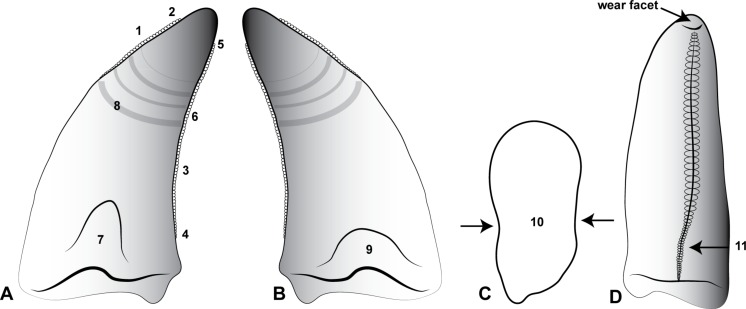
Morphological features of an *Australovenator* lateral tooth. (A) Lingual; (B) Labial; (C) asymmetrical lanceolate crown base cross-section; (D) Proximal view of distal carina. Morphological characteristics: (1) mesial carina non-denticulate (reduced denticles occasionally present apically); (2) apical denticles gradually increasing in size from their initiation on the carina then further apically decrease in size towards the tooths apex or wear facet; (3) distal denticles; (4) gradual decrease in distal denticle size towards the crown’s base; (5) gradual increase in distal denticles towards the crown’s apex; (6) average number of mid-crown denticles per 5 mm on distal carina in subadult/adult 9–15; (7) lingual depression; (8) large transversal undulations on the crown in some teeth present tenuous; (9) labial depression; (10) labial and lingual compression creating figure-of-eight morphology; (11) lingual deviation of the distal carina creating an asymmetrical lanceolate basal crown cross-section.

Where the teeth were adequately preserved, the distal carina twists lingually towards the cervix and a shallow longitudinal depression (*sensu* Hendrickx, Mateus & Araújo, 2015) is present on both the lingual and labial surfaces of the crown, similar to *Megaraptor* and *Orkoraptor* (Novas, Ezcurra & Lecuona, 2008; Porfiri et al., 2014). This combination of a lingually-twisted distal carina and the presence of longitudinal depressions convey an eight-shaped, asymmetrical lanceolate basal cross-section to each tooth (Fig. 5).

The teeth are imperfectly preserved, in which case the presence or absence of mesial carina cannot be confirmed. Serrations on the distal carina are only observable on one tooth (D4), the others being too damaged to observe (Figs. 4G–4H). This tooth preserves four symmetrical, convex, parabolic denticles per millimetre, however, the denticles are only preserved at the base of the crown where they increase in number and reduce in size towards the cervix.

The first tooth (D1) is substantially smaller than the second and third teeth and differs from the lateral teeth in having a subcircular basal cross-section being nearly as mediolaterally wide as mesiodistally long (Table 1). The anteriormost dentary teeth are similar in size to the posterior teeth, whereas the teeth in the middle of the dental arcade tend to be larger.

**Figure 6:**
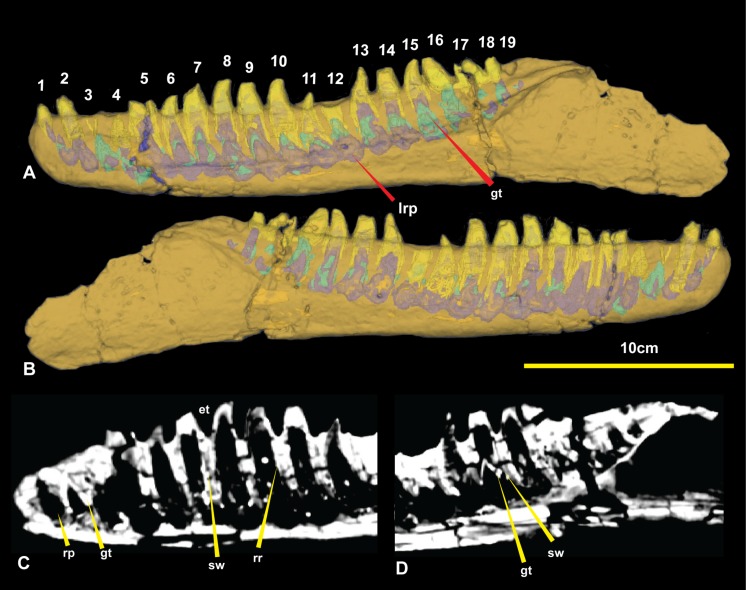
Computed tomography of right dentary. (A) Mimics render of lingual denary and internal structure; (B) Mimics render of labial dentary and internal structure; (C) CT scan of distal portion of dentary; (D) CT scan of proximal end of dentary. Abbreviations: gt, germ tooth; lrp, lingual resorption pit; rp, resorption pit; sw, socket wall; rr, residual root. Scale bar = 10 cm.

The CT data also identified the extent of erupted and developing germ teeth in all except alveolus 5, which was diagenetically fractured. Germ teeth occupy a position lingual to the base of their predecessor within the medial band in their corresponding resorption pits (Fig. 6). Most of the teeth are fully erupted; D4 and D11 are in early stages of emergence, teeth D2, D6, and D13 are not fully erupted from their respective alveoli and D16 is tilted posteriorly (distally) due to displacement by the underlying replacement tooth.

Of the missing teeth, CT scans show D2 still has a large amount of its root remaining within the alveolus indicating that it had not fully erupted. The 19th tooth has fallen out with no visible replacement or tooth root within the alveolus (Figs. 4 and 6).

### Isolated theropod teeth from the ‘Matilda’ site (AODL85)

**Figure 7:**
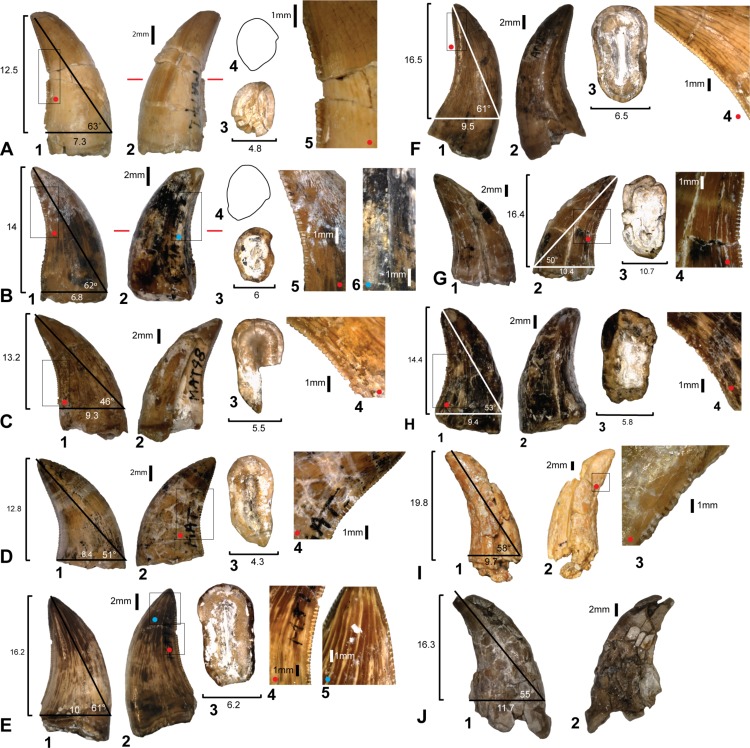
Theropod teeth discovered from Matilda Site (AODL85). (A) Premaxillary tooth (AODF831): 1, labial; 2, lingual; 3, base; 4, mid tooth cross-section; 5, denticles. (B) Premaxillary tooth (AODF822): 1, labial; 2, lingual; 3, base; 4, mid tooth cross-section; 5, mesial denticles; 6, distal denticles. (C) Dentary or maxillary teeth: (AODF823): 1, lingual; 2, labial; 3, base; 4, denticles. (D) (AODF824): 1, lingual; 2, labial; 3, base; 4, distal denticles. (E) (AODF825): 1, lingual; 2, labial; 3, base; 4, distal denticles; 5, mesial denticles. (F) (AODF826): 1, labial; 2, lingual; 3, base; 4, distal denticles. (G) (AODF829): 1, lingual; 2, labial; 3, base; 4, distal denticles. (H) (AODF828): 1, labial; 2, lingual; 3, base; 4, distal denticles. (I) (AODF827): 1, lingual; 2, labial; 3, distal denticles. (J) (AODF830): 1, lingual; 2, labial. Measurements are in millimetres.

Ten isolated teeth were collected from the ‘Matilda’ site (AODL85), which were initially assigned to the holotype of *Australovenator* (Hocknull et al., 2009). However, as pointed out by those authors, all of the teeth are broken along their bases indicating they were shed teeth and are therefore unlikely to belong to the holotype individual (Fig. 7).

Three theropod tooth morphotypes are recognised from the ‘Matilda’ site: premaxillary or mesial dentary teeth with a distinctive J-shaped basal cross-section (see Fig. 5T in Hendrickx, Mateus & Araújo, 2015); anterior dentary teeth with sub-circular bases; and labiolingually compressed lateral teeth. This distinction is supported by comparisons with the premaxillary and maxillary dentition of *Megaraptor* (Porfiri et al., 2014). These morphotypes are grouped and described here for convenience.

### Premaxillary or mesial teeth

Two teeth, AODF831 (Fig. 7A) and AODF822 (Fig. 7B) are identified as a premaxillary or mesial teeth based on their lingually oriented mesial carinae and J-shaped basal cross-sections. In AODF831, the crown is recurved and the apex shows evidence of wear. The apical wear surface is ovoid, mesio-distally oriented, and slants lingually. Below the cervix, the tooth is oval in cross-section with the labial side slightly more expanded than the lingual side. At the mid-height of the tooth, the cross-section is distinctly J-shaped due to the prominent mesial carina. The mesial carina is non-serrated, whereas the distal carina preserves 3 denticles/mm (Fig. 7A).

The denticles are symmetrical and rounded at their apex. They are largest at the mid-height of the crown and reduce in size and proportion towards the base and apex. The orientation of the mesiodistal axis of the apical denticles on the mesial carina, are perpendicular to the mesial margin. The enamel texture is completely smooth and lacks microscopic sculpturing.

The other premaxillary tooth, AODF822 (Fig. 7B) is similar to AODF831 in most respects but differs in that the mesial carina is serrated. The mesial denticles are well worn and shallower than the distal denticles and have symmetrical, rounded apices. In lateral view, the orientation of the mesiodistal axis of apical denticles on the mesial carina, are inclined apically from the mesial margin.

### Lateral teeth

**Figure 8:**
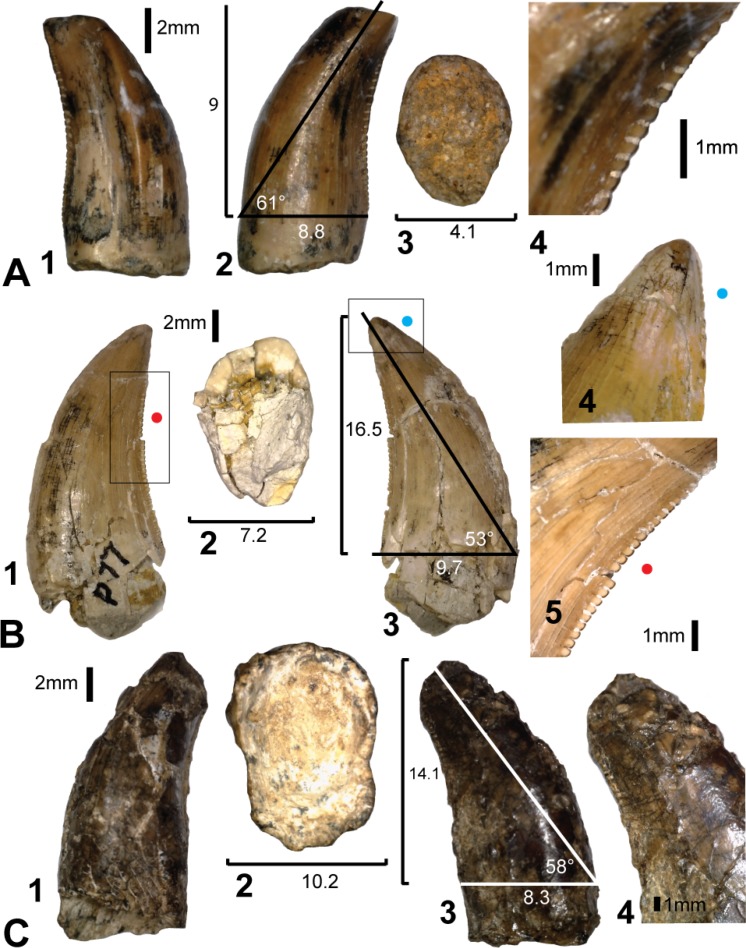
Teeth from Pegler’s Site (AODL124), Pete Site (AODL125) and Wade Site (AODL82). (A) Pegler’s Site premaxillary tooth (AODF664): 1, labial; 2, lingual; 3, base; 4, distal denticles. (B) Pete Site dentary or maxillary tooth (AODF820): 1, lingual; 2, base; 3, labial; 4, mesial denticles; 5, distal denticles. (C) Wade Site dentary or maxillary tooth (AODF819): 1, lingual; 2, base; 3, labial; 4, distal denticles. Measurements are in millimetres.

Six teeth; AODF823 (Fig. 7C), AODF824 (Fig. 7D), AODF825 (Fig. 7E), AODF826 (Fig. 7F), AODF829 (Fig. 8G), AODF828 (Fig. 8H) are identified as lateral teeth. They share a recurved and blade-like morphology with 3 denticles/mm on the distal carina. The denticles are symmetrical, parabolic, and as high as they are long with a rounded apex. The labial and lingual sides of the crown are depressed as in *Orkoraptor* (Novas, Ezcurra & Lecuona, 2008) and *Megaraptor* (Porfiri et al., 2014) (i.e., presence of longitudinal depressions), which also give the tooth a figure-of-eight basal cross-section. However, a unique lingual curvature of the distal carina near the base of the crown also confers an asymmetrically lanceolate outline in basal cross-section (Fig. 7D).

The mesial margin of the crown in lateral view is strongly convex, whereas the distal margin is gently concave in most teeth except AODF824 and AODF826 in which it is more strongly concave.

The mesial carina are generally present, however, due to the poor preservation of the mesial surface of most specimens, the presence of mesial denticles cannot be confirmed in each tooth. Mesial denticles are present in AODF825 and AODF824, although the mesial carina is present but is non-serrated in AODF826. The mesial denticles occur only in the apical region of the tooth. Where present, they are parabolic in lateral view and are distinctly shallower than the distal denticles, having a denticle height roughly half that of the denticle length. The denticles on the mesial carina are smallest apically and at the basal termination.

The average number of mid-crown denticles per 5 mm ranges from 9 to 15 on the distal carina but increases (i.e., denticles are smaller) towards both the apex and the base. The total number of denticles along the distal carina ranges between 45 and 80. The mid-crown denticles are perpendicular to the distal margin and the interdenticular spaces are narrow and less than one third of the denticle height. There are no interdenticular sulci between any of the denticles on the distal carina. Flutes, marginal undulations (*sensu* Hendrickx & Mateus, 2014) and other surface ornamentations are absent; however, AODF824 and AODF829 both have weakly developed transverse undulations. All the teeth have a smooth enamel surface.

One lateral tooth (AODF827) is baso-apically elongated and most likely represents a mesial tooth (Fig. 7I). The mesial surface is poorly preserved and the presence of mesial denticles could not be determined; however, the distal denticles possess the same morphology as the previously described lateral teeth.

### Premaxillary or mesial teeth

AODF664 (Fig. 8A) comes from Pegler’s Site (AODL124; Fig. 3) and is identified as a right premaxillary or possibly a left anterior dentary tooth based on lingual displacement of the mesial carina. It is less strongly recurved than other teeth interpreted as lateral teeth and is nearly circular in basal cross-section. The mesial carina is non-denticulate, whereas the distal carina has three symmetrically convex and apically inclined denticles per millimetre. The overall morphology is similar to premaxillary tooth AODF831 from AODL85.

### Lateral teeth

AODF820 (Fig. 8B) is a shed maxillary or dentary tooth from Pete’s Site (AODL125; Fig. 3), similar in most respects to the lateral teeth described from the Matilda Site. However, like AODF824, AODF825 and AODF826, it possesses a mesial carina with serrations restricted to the apical part of the crown. Unfortunately, they are extremely worn which obscures their original shape and density per millimetre. Like other lateral teeth from the Matilda site, the distal carina is curved lingually at its base giving the tooth an asymmetrically lanceolate basal cross section.

AODF819 (Fig. 8C) is a poorly-preserved shed maxillary or dentary tooth from Wade Site (AODL82; Fig. 3). It is recurved, missing the apical tip and has a figure-of-eight-shaped basal cross section. The morphology of the few denticles preserved on the distal carina could not be determined due to poor preservation.

## Results of the Morphometric Analysis

**Figure 9:**
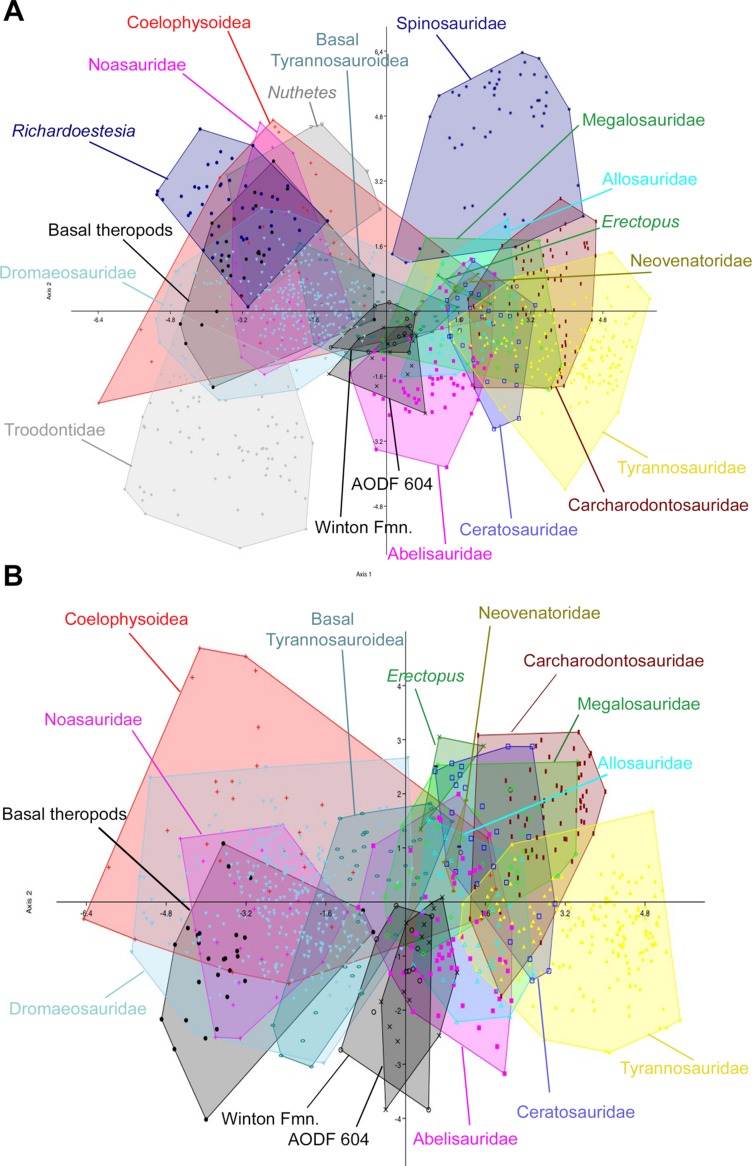
Discriminant analysis of theropod teeth from the Winton Formation showing first two principal components. (A) Unmodified complete dataset using all 20 morphotypes/clades; (B) reduced taxa dataset. Isolated teeth from the Matilda, Peglers, Wade, and Pete Sites are grouped within the ‘Winton Fmn.’ In situ teeth from the *Australovenator* holotype dentary (AODF604) were considered separately.

The initial discriminant analysis of all 21 theropod tooth morphotypes produced results similar to those of Hendrickx, Mateus & Araújo (2014), such that Troodontidae, Noasauridae, Spinosauridae, and Tyrannosauridae fall into clearly separated regions of morphospace. The first two axes constitute over 90% of the variation in the sample: Axis 1 is size dependent, being dominated roughly equally by CBW, CBL, and CH, whereas axis 2 is dominated by DC (Table 3). *Australovenator* and the isolated Winton teeth closely overlap one another in morphospace; however, they can be differentiated from Carcharodontosauridae, Ceratosauridae, Megaraptora, Neovenatoridae, Noasauridae, Spinosauridae, Troodontidae, and Tyrannosauridae as well as *Erectopus, Nuthetes, Piatnitzkysaurus*, and *Richardoestesia* (Fig. 9). The reduced taxa dataset did not provide additional resolution with the exception of non-neotheropod theropods, which could be differentiated from both *Australovenator* and the isolated Winton teeth. The first two axes constitute 96% of the total variation in the sample: Axis 1 is interpreted again as overall size, dominated by CBW, CBL, and CH, whereas axis 2 is dominated by CHR (Table 4).

**Table 3:** Results from PCA of unmodified dataset of all teeth showing relative importance of first four principle components (eigenvalue) and relative loadings for each principle component.

Variable	PC1	PC2	PC3	PC4
% variance	67.95	22.86	4.366	2.919
CBL	0.1365	−0.026275	0.036301	0.0093922
CBW	0.15602	−0.018365	−0.032585	0.02698
CH	0.14155	−0.0030737	0.046427	0.025165
MC	−0.026084	0.047247	0.0028644	0.091233
DC	−0.043486	0.080855	−3.12E−05	−0.033596
CBR	−0.019393	−0.0074067	0.068029	−0.019928
CHR	−0.014362	0.012764	0.076525	0.0040834

**Table 4:** Results from PCA of the reduced taxa dataset showing relative importance of first four principle components (eigenvalue) and relative loadings for each principle component.

Variable	PC1	PC2	PC3	PC4
% variance	88.33	7.533	2.732	2.457
CBL	0.13655	0.041797	−0.022166	−0.0056166
CBW	0.1527	−0.010319	0.018813	0.036054
CH	0.13525	0.056308	−0.011255	0.068751
MC	−0.036269	0.037686	0.02148	−0.041604
DC	−0.054281	0.028687	0.064881	−0.032239
CBR	−0.015893	0.05184	−0.040561	−0.043878
CHR	−0.016962	0.065688	−0.028575	0.025993

**Figure 10:**
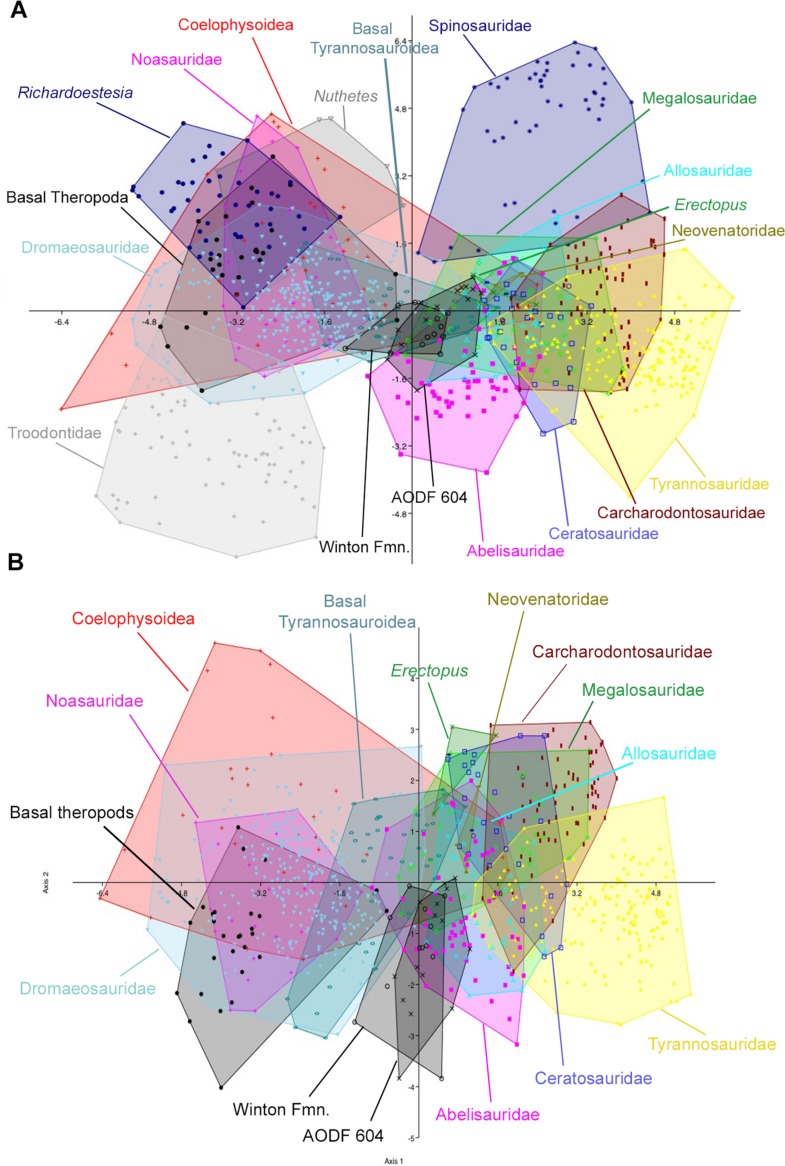
Discriminant analysis of theropod teeth using reconstructed crown height for *Australovenator*, and showing first two principal components. (A) Complete dataset using all 20 morphotypes/clades; (B) reduced taxa dataset. Isolated teeth from the Matilda, Peglers, Wade, and Pete Sites are grouped within the Winton Formation. In situ teeth from the *Australovenator* holotype dentary (AODF604) were considered separately.

The second set of analyses, using reconstructed crown height for the teeth of *Australovenator*, produced results nearly identical to the first two analyses. In the analysis of all 21 theropod tooth morphotypes, the first two axes constitute over 90% of the variation in the sample: Axis 1 is size dependent, and is dominated roughly equally by CBW, CBL, and CH, whereas axis 2 is dominated by DC and CBR (Table 5). In the reduced taxa dataset, axes 1 and 2 constitute over 94% of the total variation. Axis 1 is dominated by CBW, CBL, and CH, whereas axis 2 is dominated by CHR, CBR, and CH (Table 6; See Stat Table S1) for more detailed results) (Fig. 10).

**Table 5:** Results from PCA of the modified dataset using reconstructed crown height showing relative importance of first four principle components (eigenvalue) and relative loadings for each principle component.

Variable	PC1	PC2	PC3	PC4
% variance	68.6	22.82	4.408	2.88
CBL	0.13653	−0.025762	0.036401	1.30E−02
CBW	0.15614	−0.017603	−0.036913	0.020019
CH	0.14151	−0.0036217	0.037218	0.018323
MC	−0.026177	0.047515	0.0020782	0.10006
DC	−0.043658	0.081375	0.0068202	−0.027775
CBR	−0.019477	−0.0076419	0.07274	−0.0090921
CHR	−0.014511	0.011438	0.071475	0.0045747

**Table 6:** Results from PCA of the modified reduced taxa dataset. Reconstructed crown heights were used showing relative importance of first four principle components (eigenvalue) and relative loadings for each principle component.

Variable	PC1	PC2	PC3	PC4
% variance	87.13	7.514	2.818	1.594
CBL	0.1364	0.044309	−0.019131	−0.0054598
CBW	0.15262	−0.014378	0.013458	0.030672
CH	0.13506	0.051802	−0.019078	0.068844
MC	−0.03626	0.037265	0.03164	−0.050864
DC	−0.054256	0.028492	0.069421	−0.013564
CBR	−0.015955	0.058067	−0.03037	−0.042631
CHR	−0.017075	0.065331	−0.030192	0.028222

In all four analyses, the first two axes show considerable overlap between *Australovenator* and the isolated Winton teeth, suggesting that the two groups are not morphometrically distinguishable. Both groups also overlap to varying extents with Abelisauridae, Allosauridae, Coelophysoidea, Megalosauridae and basal Tyrannosauroidea. 81.8% and 90.1% of *Australovenator* teeth were correctly classified in the first set of analyses using the full (i.e., all 21 taxa) and reduced taxa datasets, respectively (Table S1). When actual crown height was substituted for reconstructed crown height (for *Australovenator*), this value fell to 50% and 33.3% for the full and reduced taxa datasets, respectively. Of the misclassified *Australovenator*teeth, 25% and 41.7% (for the full and reduced taxa datasets, respectively) were grouped with the isolated Winton teeth. Of the isolated Winton teeth, 76.9% of teeth were correctly identified in the first set of analyses using both the full (i.e., all 21 taxa) and reduced taxa datasets, whereas only 60% were correctly identified in the second test using reconstructed crown height in both the full and reduced taxa datasets. Of the misclassified isolated teeth, 15.4% were identified as *Australovenator* in the first two analyses, whereas 13.3% were identified as *Australovenator* in the analyses using reconstructed crown height.

## Discussion

The preponderance of megaraptorids in the Winton Formation is unusual among penecontemporaneous Gondwanan theropod faunas where abelisauroids and carcharodontosaurids make up a considerable proportion of the fauna in terms of both diversity and total abundance (Weishampel et al., 2004; Novas, 2009; Novas et al., 2013). However, new theropod discoveries within Australia in areas such as Lightning Ridge, NSW (Bell et al., 2015), and coastal Victoria (Benson et al., 2012) are suggesting a dominance of megaraptorans Australia wide. In addition, the repeated co-occurrence of isolated theropod teeth alongside associated and disarticulated sauropod remains in the Winton Formation suggests sauropods formed a considerable part of the megaraptorid diet.

Morphometric analyses of all theropod teeth known from the Winton Formation found that, in general, isolated crowns could not be distinguished from the *in situ* dentary teeth of *Australovenator* corroborating the qualitative evidence that they are assignable to *Australovenator wintonensis*. The nearly identical results between the modified (i.e., using reconstructed crown height) and the unmodified datasets, suggests that crown height can be accurately estimated in *Australovenator* using the methods described here without altering the statistical results; however, we recommend caution when applying this to other taxa. Interestingly, all Winton teeth were distinguishable from Neovenatoridae (*Neovenator*) and other Megaraptora (*Aerosteon, Fukuiraptor*), although the sample size from the latter two groups is small (three teeth for *Neovenator*; one tooth each for *Aerosteon* and *Fukuiraptor*). Increased sampling of *Megaraptor* based on skull described by Porfiri et al. (2014) would help improve this resolution.

Four of the isolated teeth (AODF823, AODF829, AODF828 and AODF820) (Fig. 8) possess mesial carinae with reduced serrations although the majority of teeth had unserrated mesial carinae. Although mesial denticles were not observed on the *in situ* teeth, we cannot entirely rule out their presence as many of the teeth were too incompletely preserved. The denticles visible on the single *in situ* tooth have the same morphology as those preserved in the isolated teeth (Fig. 4H). The teeth within the dentary also share a similar crown basal cross-section with the isolated lateral teeth demonstrating an asymmetrical lanceolate and figure-of-eight shape.

The identification of premaxillary teeth referrable to *Australovenator* cannot be confirmed without the discovery of a premaxilla with *in situ* teeth. However their similarity to the recently-described *Megaraptor* premaxillary teeth (see Fig. 2 in Porfiri et al., 2014) coupled with quantitative and qualitative evidence presented here supports the assignment of the isolated premaxillary teeth to *Australovenator* sp.

In comparison with other theropod groups, the teeth described here lack enamel undulations and interdenticular sulci that are characteristic of some carcharodontosaurids (Currie & Azuma, 2006; Novas et al., 2013). They also differ from the teeth of some abelisauroids in lacking interdenticular sulci and having a recurved, rather than straight distal carinae (Bittencourt & Kellner, 2002; Smith, 2007). They are further differentiated from spinosaurids, which have sub-circular basal cross sections and lack denticles on both mesial and distal carinae (*Angaturama, Irritator, Spinosaurus*) or have fine denticles (*Baryonyx, Suchomimus*) (Hendrickx, Mateus & Araújo, 2015). Conversely, mesial denticles that are restricted to the apical part of the crown have been described for some megaraptorids and megalosaurids (Hendrickx, Mateus & Araújo, 2015).

Based on the combined evidence from the *in situ* dentary teeth and assigned isolated teeth, the dentition of *Australovenator* demonstrates a modest degree of heterodonty that is also demonstrated in *Megaraptor* (Porfiri et al., 2014), basal tyrannosauroids (e.g., *Proceratosaurus*; Rauhut, Milner & Moore-Fay, 2010), and tyrannosaurids (Smith, 2005; Buckley et al., 2010). In basal cross-section, the first dentary tooth of *Australovenator* is subcircular whereas all other dentary teeth are figure-of-eight shaped and asymmetrically lanceolate in basal cross section (due to the presence of a labial and lingual longitudinal depression that extends through to the root). This general arrangement appears to be mirrored in the upper dental arcade of *Megaraptor* (small premaxillary teeth and large eight-shaped lateral teeth (Porfiri et al., 2014) and the assigned isolated teeth of *Australovenator*, which may imply a functional separation between mesial and distal teeth (Reichel, 2010; Reichel, 2012).

Interestingly, the premaxillary teeth of *Australovenator* and *Megaraptor* both have a J-shaped basal cross section (see Fig. 2 in Porfiri et al., 2014). This condition is considered widespread amongst Avetheropoda but reaches a U-shape in Tyrannosauridae (Hendrickx & Mateus, 2014) and the two megaraptorans. Until recently, the characterisation of megaraptorid dentition was problematic. A single tooth described in connection with the holotype of *Aerosteon* Sereno et al. (2008), is probably abelisaurid (supplementary information in Novas et al., 2013). In both *Orkoraptor* and in the original material described for *Australovenator* (Hocknull et al., 2009), teeth are represented by incomplete or shed tooth crowns, which, despite their unique morphologies (compared to most theropods), casts some doubt as to their relationship with the associated skeletal material. The new dentary of *Australovenator* permits robust characterisation of megaraptorid dentary teeth and compares well to the recently-described premaxillary and maxillary dentition of *Megaraptor* (Porfiri et al., 2014). The *in situ* and isolated teeth of *Australovenator* confirm the megaraptorid combination of serrated distal and non-serrated (or reduced serrate) mesial carinae, and lateral teeth with an eight-shaped basal cross section (i.e., presence of longitudinal depressions on labial and lingual surfaces (Fig. 5) (Novas, Ezcurra & Lecuona, 2008), neither of which are present in basal megaraptorans (*Fukuiraptor*). The combination of eight-shaped teeth with reduced mesial denticles has also been observed in isolated teeth from the lower–middle Aptian Wonthaggi Formation in Victoria, thus supporting their referral to Megaraptoridae (Benson et al., 2012).

The absence or reduction of mesial denticles has been cited as a feature linking megaraptorans with coelurosaurs (Novas, Ezcurra & Lecuona, 2008; Novas et al., 2013); however, this assumption was originally based on a small number of isolated teeth collected alongside the holotype of *Orkoraptor*. This has since been verified from *in situ* teeth in *Megaraptor* (Porfiri et al., 2014) and we confirm that some isolated teeth assignable to *Australovenator* (AODF822, AODF825) retain reduced denticles on the apical part of the mesial carina, which we here attribute to intra-jaw and/or individual variation. As pointed out by Hendrickx, Mateus & Araújo (2015), Novas, Ezcurra & Lecuona (2008) and Novas et al. (2013) the absence of mesial denticles is relatively common in mesial teeth (*Eoraptor*, *Herrerasaurus*, *Aviatyrannis*, *Ornitholestes*, many compsognathids, and some troodontids and dromaeosaurids). In lateral teeth, this features is, indeed, present in compsognathids and “deinonychosaurs,” but also in some basal coelurosaurs/maniraptoriformes like *Ornitholestes*, *Zuolong*, and *Aorun*, the juvenile megalosaurid *Sciurimimus*, and the basal alvarezsauroid *Haplocheirus* (C Hendrickx, pers. comm., 2015).

Lateral teeth with a figure-of-eight-shaped basal cross section (i.e., presence of longitudinal depressions on basal labial and lingual surfaces) (Novas, Ezcurra & Lecuona, 2008) are characteristic of *Orkoraptor* and *Megaraptor* (Porfiri et al., 2014); however, they are also identified here for *Australovenator* suggesting this feature may be a synapomorphy of Megaraptoridae, convergently acquired in some deinonychosaurians (e.g., *Deinonychus, Saurornitholestes*) and some tyrannosauroids such as *Proceratosaurus* (Rauhut, Milner & Moore-Fay, 2010) and *Alioramus* (Brusatte et al., 2012).

The lingual twist at the base of the distal carina is a feature also shared with dromaeosaurs and megaraptorans including potential megaraptorid teeth from the Wonthaggi Formation of Victoria (Benson et al., 2012).

The teeth of *Fukuiraptor* (Azuma & Currie, 2000; Currie & Azuma, 2006; Novas et al., 2013) differ markedly from *Australovenator* and other megaraptorans: They are strongly mediolaterally compressed, bladelike teeth with well-developed caudae and denticulate mesial and distal carinae on both premaxillary and non-premaxillary teeth (Currie & Azuma, 2006). Interestingly, the maxillary teeth of a juvenile *Megaraptor* have a braided enamel texture (C Hendrickx, pers. comm., 2015), non-serrate mesial carinae and lack interdenticular sulci altogether (Porfiri et al., 2014). The smooth enamel texture, reduced mesial serrations, and lingually twisted distal carina in *Australovenator* may indicate subtle variations between *Megaraptor* and *Australovenator* but confirmation of these differences would require the discovery of additional material from both taxa.

## Conclusions

The newly-discovered dentary of *Australovenator* is the most complete megaraptorid dentary yet known. Morphological features of the dentary include: an elongate, shallow profile in lateral aspect; an elliptical anterior region in sagittal view; a reduced or absent symphyseal facet, and; nineteen aveoli. *Australovenator* presents modest heterodonty in the lower jaw: the first dentary tooth is ovoid in basal cross-section whereas all other lateral teeth are mediolaterally compressed; possess lingual and labial depressions, and have a distal carina that curves lingually close to the cervix. Mesial denticles are typically absent but may occur close to the apex. Megaraptorid teeth can be characterised as having figure-of-eight basal cross-sections, serrate distal carinae and non-serrate mesial carinae (although serrations may rarely be present on the apical tip of the crown). Enamel wrinkles and prominent caudae, which are common in other Gondwanan theropods (abelisauroids and carcharodontosaurids), are also absent in the teeth of *Australovenator* and other megaraptorids.

At present, most of the isolated theropod teeth so far collected from the Winton Formation can be confidently assigned to *Australovenator* based on morphological and morphometric evidence.

## Supplemental Information

10.7717/peerj.1512/supp-1 Figure S1 Right dentary of *Australovenator wintonensis* The holotype AODF 604 right dentary of *Australovenator wintonensis.*

10.7717/peerj.1512/supp-2 Figure S2 Left dentary of *Australovenator wintonensis* AODF 604 The holotype left dentary of *Australovenator wintonensis*: (A, B) Dorsal; (C, D) Lingual; (E, G) Labial. Abbreviations: Mg, Meckelian groove; sym, symphysis. Scale bar = 10 cm.

10.7717/peerj.1512/supp-3 Figure S3 Left dentary of *Australovenator wintonensis*

10.7717/peerj.1512/supp-4 Table S1 Full and reduced data sets. Full (i.e., all 20 taxa) and reduced taxadatasets

## Additional Information and Declarations

### Data Availability

The following information was supplied regarding data availability: The research in this article did not generate any raw data.
