# Supplementary figures and images for: The dentary of Australovenator wintonensis (Theropoda, Megaraptoridae); implications for megaraptorid dentition
Source: PeerJ. 2015 Dec 15;3:e1512. doi: 10.7717/peerj.1512 (PMC4690360; doi:10.7717/peerj.1512)

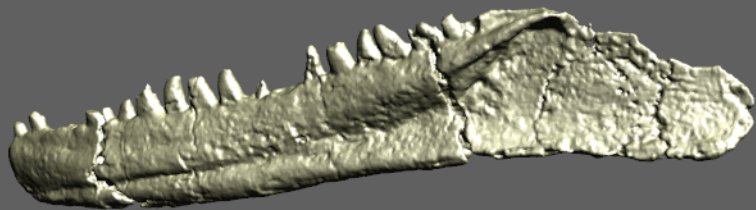

Supplement: Figure S1 — The holotype AODF 604 right dentary of Australovenator wintonensis. [file peerj-03-1512-s001.pdf]

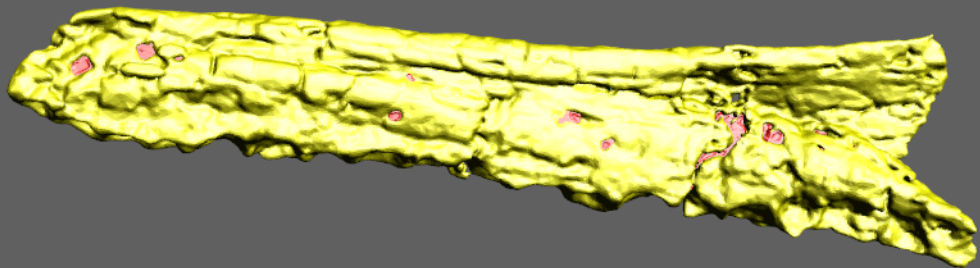

Supplement: Figure S2 — The holotype left dentary of Australovenator wintonensis: (A, B) Dorsal; (C, D) Lingual; (E, G) Labial. Abbreviations: Mg, Meckelian groove; sym, symphysis. Scale bar = 10 cm. [file peerj-03-1512-s002.pdf]
