# Supplementary material for: The dentary of Australovenator wintonensis (Theropoda, Megaraptoridae); implications for megaraptorid dentition
Source: PeerJ. 2015 Dec 15;3:e1512. doi: 10.7717/peerj.1512 (PMC4690360; doi:10.7717/peerj.1512)
Supplement: Supplementary file 2 [file peerj-03-1512-s004.docx]

	Axis 1	Axis 2	Axis 3	Axis 4	Axis 5	Axis 6	Axis 7	Axis 8	Axis 9	Axis 10	Axis 11	Axis 12	Axis 13	Axis 14	Axis 15	Axis 16
CBL	0.1364	0.044309	-0.019131	-0.0054598	0.010839	-0.0029525	0.033148	0.00E+00	2.5551E-314	-1.26E-243	-0.019245	0.099141	-0.076423	0.033379	-0.03716	0.020853
CBW	0.15262	-0.014378	0.013458	0.030672	0.039801	0.036232	-0.0097942	0.00E+00	1.3008E-314	1.19E-244	0.0079014	0.080597	-0.068013	0.0060458	-0.11699	0.00079226
CH	0.13506	0.051802	-0.019078	0.068844	0.024174	0.029638	0.042897	0.00E+00	4.8615E-314	1.02E-243	-0.023101	0.1326	-0.063928	0.031627	-0.061363	0.033857
MC	-0.03626	0.037265	0.03164	-0.050864	0.092236	-0.013222	-0.0038032	0.00E+00	6.1649E-315	1.59E-244	-0.0018743	-0.033737	0.038605	0.087591	0.015236	-0.042596
DC	-0.054256	0.028492	0.069421	-0.013564	-0.028673	-0.019103	0.00020897	0.00E+00	2.2134E-315	7.30E-245	-0.0037373	-0.030723	-0.000347	0.014346	0.044155	-0.0685
CBR	-0.015955	0.058067	-0.03037	-0.042631	-0.032314	0.053878	-0.0084638	0.00E+00	4.5852E-315	4.65E-244	0.0020813	0.027029	-0.0054794	-0.031894	0.07771	-0.013085
CHR	-0.017075	0.065331	-0.030192	0.028222	-0.01667	-0.011752	-0.063441	0.00E+00	1.891E-314	-3.95E-244	0.0050825	0.063231	0.067129	-0.022461	0.030224	-0.023976

Confusion Matrix

	1	2	3	4	5	6	7	8	10	11	12	13	14	16	19	20	21	Total
1	21	2	0	2	0	0	0	0	0	0	0	2	0	1	0	0	0	28
2	3	19	0	1	0	0	0	0	0	4	0	1	0	3	0	0	0	31
3	0	0	4	0	4	2	4	6	2	0	0	0	2	0	0	0	2	26
4	5	1	0	11	0	0	0	0	0	0	0	1	0	6	0	0	0	24
5	0	0	5	0	32	0	1	1	5	4	0	3	0	0	3	0	1	55
6	0	0	0	0	0	2	1	0	0	0	0	0	0	0	0	0	0	3
7	0	0	0	0	0	0	2	0	0	0	0	0	0	0	0	0	0	2
8	0	0	7	0	2	1	3	4	3	2	4	4	0	0	0	0	3	33
10	0	0	0	0	3	0	1	5	18	2	0	0	0	0	1	0	1	31
11	0	0	0	0	0	0	1	0	0	2	0	0	0	0	0	0	0	3
12	0	0	2	0	1	0	2	0	0	8	48	0	1	0	1	0	1	64
13	1	0	1	3	0	0	0	2	0	4	0	20	0	0	0	5	3	39
14	0	0	1	0	2	0	0	2	11	5	5	0	135	0	3	0	0	164
16	33	23	0	33	0	2	0	0	0	0	0	32	0	154	1	15	4	297
19	0	0	0	0	1	0	0	0	0	1	0	2	0	0	9	2	0	15
20	0	0	0	0	1	0	0	0	0	0	0	2	0	0	5	4	0	12
21	0	0	0	0	0	0	0	0	0	0	0	0	0	0	0	0	2	2
Total	63	45	20	50	46	7	15	20	39	32	57	67	138	164	23	26	17	829

% Variance

	Axis 1	87.13%
	Axis 2	7.514
	Axis 3	2.818
	Axis 4	1.594
